# New targets for therapy: antigen identification in adults with B-cell acute lymphoblastic leukaemia

**DOI:** 10.1007/s00262-020-02484-0

**Published:** 2020-01-22

**Authors:** Stephanie Jordaens, Leah Cooksey, Laurie Freire Boullosa, Viggo Van Tendeloo, Evelien Smits, Ken I. Mills, Kim H. Orchard, Barbara-ann Guinn

**Affiliations:** 1 Department of Biomedical Sciences, University of Hull, Cottingham Road, Hardy Building, Room 111, Hull, HU7 6RX UK; 2 Laboratory of Experimental Hematology, Vaccine and Infectious Disease Institute, University of Antwerp, Antwerp, Belgium; 3 Centre for Oncological Research, University of Antwerp, Antwerp, Belgium; 4 Centre for Cancer Research and Cell Biology, Queens University Belfast, Lisburn Road, Belfast, UK; 5 Department of Haematology, University Hospital Southampton NHS Foundation Trust and University of Southampton, Southampton, UK

**Keywords:** Immunotherapy, Tumour antigens, Survivin, BMX, B-cell acute lymphoblastic leukaemia, PIVAC19

## Abstract

Acute lymphoblastic leukaemia (ALL) in adults is a rare and difficult-to-treat cancer that is characterised by excess lymphoblasts in the bone marrow. Although many patients achieve remission with chemotherapy, relapse rates are high and the associated impact on survival devastating. Most patients receive chemotherapy and for those whose overall fitness supports it, the most effective treatment to date is allogeneic stem cell transplant that can improve overall survival rates in part due to a ‘graft-versus-leukaemia’ effect. However, due to the rarity of this disease, and the availability of mature B-cell antigens on the cell surface, few new cancer antigens have been identified in adult B-ALL that could act as targets to remove residual disease in first remission or provide alternative targets for escape variants if and when current immunotherapy strategies fail. We have used RT-PCR analysis, literature searches, antibody-specific profiling and gene expression microarray analysis to identify and prioritise antigens as novel targets for the treatment of adult B-ALL.

## Introduction

Acute lymphoblastic leukaemia (ALL) is a clonal malignant disease that originates in a single B- or T-lymphocyte progenitor and is characterised by diverse cytogenetic and molecular abnormalities. The uncontrolled proliferation and accumulation of these leukaemic cells in the bone marrow results in the suppression of normal haematopoiesis and infiltration of diseased cells into various extramedullary sites, such as the liver, spleen, lymph nodes, thymus, meninges and gonads. ALL is currently the most common malignancy in children worldwide and the majority of patients with ALL have the B-cell sub-type [1]. However, for adults, the global incidence of B-ALL is around 1–5 per 100,000 persons per year, making it a rare disease that can cause death within a few weeks or months if left untreated.

## Recent developments in treatment options

Different genetic alterations, such as aberrant expression of proto-oncogenes, chromosomal translocations, hypodiploidy and hyperdiploidy, all contribute to the leukaemic transformation of haematopoietic stem cells or their committed progenitors by changing cellular functions, such as normal proliferation, differentiation and apoptosis [2]. These primary oncogenic events are often insufficient by themselves to cause leukaemia and require secondary cooperative mutations. In B-ALL, the most frequent and clinically relevant cytogenetic abnormality, accounting for up to 50% of adult B-ALLs, is the Philadelphia chromosome (Ph), a *t* (9;22)(q34;q11) translocation that leads to the expression of the BCR–ABL fusion protein. However, studies by our group do not focus on this type of B-ALL due to existing effective tyrosine kinase inhibitor (TKI) therapies, such as Imatinib (recently reviewed in [3]) and their subsequent second- and third-generation inhibitors. Other cytogenetic rearrangements include mixed lineage leukaemia (MLL) rearrangements found in 20% of all cases of ALL [1]. The MLL gene is involved in more than 50 fusions, which may play a role in transformation of bone marrow cells through the regulation of HOX genes. The most frequent gene mutations found in B-ALL patient samples are those that affect the Ikaros family zinc finger protein 1 (IKZF1), a transcription factor and regulator of normal lymphoid development and differentiation [2].

Treatment of ALL is divided into three phases: remission induction, consolidation and maintenance therapy and usually takes 2–3 years, of which the maintenance phase is the longest [4]. Chemotherapy is usually preceded by the administration of steroids, while chemotherapy itself uses cytotoxic drugs such as asparaginase, cyclophosphamide, doxorubicin, methotrexate and vincristine to destroy the cancer cells. Standard treatment methods can be particularly toxic for older (> 65 years) ALL patients. Therefore, it is even more difficult to determine the best therapy for these patients and often requires the development of personalised treatment regimens [4]. Following chemotherapy there are molecular therapeutic agents including TKIs that inhibit Fms-like tyrosine kinase-3 (FLT3), farnesyl transferase, DNA methyltransferase, histone deacetylase, mammalian target of rapamycin (mTOR), gamma-secretase, proteasome and cyclin-dependent kinases. In addition, BCL2 antisense therapy and heat-shock protein antagonists [5] are undergoing preclinical or early clinical development. There are several new treatment options under investigation in clinical trials for refractory/relapsed (R/R) B-ALL [4] including monoclonal antibodies (mAbs), antibody–drug conjugates (ADC), bispecific T-cell engager (BiTE) and chimeric antigen receptor (CAR) T-cell therapy.

The prognosis for patients with B-ALL depends on a number of factors including age/fitness, stage and cytogenetic abnormalities. Around 80–90% of ALL patients will achieve a first remission but many will relapse and overall survival (OS) remains low in adults (30–40%). The best treatment option for B-ALL patients to date has been allogeneic-haematopoietic stem cell transplantation (HSCT) [6] in first complete remission but it has limitations, due to the toxicities associated with the treatment and associated high-treatment-related mortality rates. Donor leukocyte infusions (DLIs) are already used to boost the graft-versus-leukaemia effect in patients and there is a balance required to achieve a minimal but necessary concurrent graft-versus-host disease. Immunotherapy can also be used to boost the anti-tumour activity of the immune response and ideally reduce tumour load during first remission, delaying if not preventing, relapse.

## Immunotherapy for adult B-ALL patients

The most promising agents currently available are those directed against cell membrane antigens, such as CD19, CD20, CD22 and CD52 and these signalling pathways are also important in the control of cell proliferation and apoptotic responses [3]. Currently, paediatric-inspired regimens are being tested on adolescents and young adult patients and lead to improvements in event-free survival (EFS) and OS rates. Some studies include older patients and consistently demonstrate significant improvements in EFS and OS rates, ranging from 60 to 80%, compared to historical controls. These treatments would make it possible to avoid HSCT in elderly patients and the associated risks. The biggest challenge now is to determine the maximum age limit for these treatments, taking into account the age-related and treatment-related increase in toxicities [3].

## Naked MAbs

MAbs were developed against specific cell surface antigens on the majority of diseased cells from B-ALL patients (CD19, CD20, CD22 and CD52) and exert their function through antibody-dependent cytotoxicity, complement-dependent cytotoxicity and direct induction of apoptosis [5, 7]. These target antigens are often expressed by healthy tissues as well as leukaemia cells, which reduces the cytotoxic selectivity of the treatment [5]. CD20 is a surface marker of B-lineage lymphocytes and is present on cells from 25% of patients with pre-B ALL and nearly all mature ALL cells [7]. Rituximab, a humanised anti-CD20 antibody, was the first mAb approved for therapeutic use [5]. However, several limitations were discovered with Rituximab and second-generation anti-CD20 mAbs were developed, namely Ofatumumab and Obinutuzumab [7]. Ofatumumab targets a different CD20 epitope, and is more potent than Rituximab, as efficacy has been enhanced by glycol engineering the Fc-region sugar residues of the humanised anti-CD20 mAb. Epratuzumab, a humanised anti-CD22 antibody, has appreciable anti-tumour activity and a good safety profile while Alemtuzumab is a fully humanised mAb against CD52. CD52, a cell surface glycoprotein, is involved in T-cell activation and is also expressed on pre-B ALL cells [2, 5, 7].

## ADC

The ubiquitous B-cell marker, CD19, is an unsuitable target for naked mAbs because it internalises upon binding, reducing the potential to activate complement [7]. Therefore, ADCs against CD19 were developed. The first ADC against CD19 was SAR3419, which fused a humanised anti-CD19 antibody and maytansin, a cytotoxic agent that disrupts tubule formation. Another ADC targeting CD19 was SGN-CD19A which fused an anti-CD19 antibody with the microtubule-disrupting agent monomethyla-uristain F (MMAF) [7]. Another potential target on B-ALL is CD22 which is expressed on leukaemic blasts in 90% of patients with pre-B ALL and mature ALL. Inotuzumab ozogamicin was developed to target CD22 and couples an engineered humanised monoclonal immunoglobulin G4 antibody against CD22 with the potent cytotoxic agent calicheamicin [7], a DNA-binding macromolecule.

## Inotuzumab ozogamicin

Phase I/II and III trials have shown promising results with high numbers of patients achieving minimal residual disease negative status and impressive rates of complete remission [3, 8, 9]. High response rates allow more patients to proceed to HSCT [9], it is better than standard salvage chemotherapy in R/R patients, and has efficacy when used in combination with low-dose chemotherapy for older adults with newly diagnosed ALL (NCT0371630) [10] but without additional toxicity. A concern with inotuzumab ozogamicin has been the incidence of veno-occlusive disease [8–10] and liver-related toxicities [7, 9, 10] that can be ameliorated by careful patient management. However, the safety analysis showed that patients treated with inotuzumab ozogamicin had less thrombocytopenia and less febrile neutropenia than those on standard therapy [9].

## BiTE

BiTEs conjugate two mAbs recognizing leukaemic cells and cytotoxic T lymphocytes (CTLs), a CD3- and a CD19-binding part, and direct CTLs against malignant B cells. Blinatumomab was the first-approved BiTE to be used to treat ALL [7, 11, 12]. Phase I/II and phase III trials conducted with blinatumomab described better results for patients in terms of complete remission, minimal residual disease responses, response rates and survival [9]. Blinatumomab has been associated with some adverse events, such as neutropenia, infections, liver-associated disorders, neurological events, cytokine release syndrome (CRS), infusion reactions and lymphopenia in B-ALL patients. Although these side effects can be serious, they are reversible with the administration of steroids and temporary withdrawal of the drug [9]. A major disadvantage of treatment with blinatumomab is the administration of the agent that requires continuous infusion for 4 weeks with weekly changes of infusion bags. Initially it was very costly, between 90,000 and 110,000 US dollars per cycle, but costs have significantly fallen in recent times.

## CAR T

CAR T cells are fusion proteins and consist of a mAb single-chain variant fragment (scFV), a hinge domain, transmembrane domain and intracellular T-cell activation domains often in combination with costimulatory domains [1, 7]. They bind to cell surface antigens and have the potential to enter the cerebrospinal fluid [1, 7]. CD19-targeting CAR T cells have already proved to be powerful immunotherapy agents for B-ALL patients [2, 7] despite differences in each trial in CAR design, conditioning regimens, leukaemia load, patient age, T-cell manufacturing and T-cell dosages [3, 7, 9, 13]. All clinical trials reported toxicities including on-tumour off-target effects, CRS and neurotoxicity (referred to as immune effector cell (IEC)-associated neurotoxicity or ICANS). Off-target side effects such as CRS and neurotoxicity are due to cytokine release at the time of CAR-T activation causing toxicity to non-target organs; on-target/off-tumour side effects are due to B-cell depletion with hypogammaglobulinaemia and are associated with an increased risk of infection. Critically, the establishment of guidelines to manage the toxicity associated with CAR-based adoptive immunotherapy and the durability of responses by patients are yet to be determined.

## Immune checkpoint inhibitors

Immune checkpoint inhibitors have been shown to enhance the outcomes of CD19 CAR T-cell therapies for children with relapsed B-ALL and are now entering the arena of adult B-ALL treatments. Drugs such as Ipilimumab, a fully humanised mAb that blocks the immunosuppressive signals by CTLA-4 and Pembrolizumab, a programmed cell death protein-1 (PD-1) inhibitor, are being used in clinical trials with evidence indicating that the sooner Pembrolizumab is added to treatment with CAR T cells, the more likely it will help prolong remission.

## Biomarkers for response to therapy

Outcomes for patients with R/R disease are improving thanks to the rapid development of naked mAbs, ADCs, BiTEs and adoptive T-cell therapies. The majority of B-ALL patients express the necessary surface markers targeted by most immunotherapies. Although the efficacy of these therapies is increasing, they are not without toxicity and remain expensive. Biomarkers that indicate efficacy of immunotherapy and patient responses continue to be sought. Current examples include polyfunctional CAR-T cells that may be a surrogate biomarker for treatment efficacy, with Melan-A recognised by T cell 1 (MART1)-specific TCR-engineered T cells revealing an association between TNFα and IFNγ secretion and melanoma patients with a delay in their time to disease-related relapse [14]. In chronic lymphocytic leukaemia (CLL) highly functional CAR T cells were enriched in memory-related genes including STAT3 and IL-6 signatures, while non-responders had upregulated genes associated with effector differentiation, glycolysis, exhaustion and apoptosis [15]. Of note, CD27^+^PD-1^−^CD8^+^ CAR T cells with elevated IL-6 receptor expression correlated with tumour control and response to treatment in this patient group.

We need to identify new targets for treatments for adult B-ALL patients who are ineligible for existing therapies. Biomarkers that help identify the patients who will not respond to existing treatments are essential to enhance quality of life and to identify the patient groups for whom new targets for therapy are especially required.

## New targets for immunotherapy: insights into the biological basis of disease

Our research has involved the identification of antigens for the immunotherapy of diseases that lack suitable or sufficient targets. Although the methods we use identify antigens via antibody responses in patient sera, we and others have shown that the antigens identified by antibody responses are also recognised by T cells in the context of MHC [16]. This is likely because activation of normal B cells improves antigen presentation and induces specific CD4^+^ and CD8^+^ T-cell responses. Notable benefits of our research have been that these antigens have also helped us better understand the molecular mechanisms underlying early-stage disease processes [17] and identified biomarkers for survival [18].

Tumour antigens can be subdivided into a number of different ways. One is to group tumour antigens based on their expression pattern and/or disease-associated aberration; however, few antigens fit into just one group. Antigen groupings include tumour-specific antigens that are exclusively expressed in tumour cells and are typified by cancer-testis antigens (CTAs), which are found in tumour cells and immune privileged (MHC class I negative) tissues such as the placenta and testis. The next groups encompass tumour-associated antigens (TAAs) that are mutated (neoantigens) (reviewed in [19]), differentiation products and/or overexpressed antigens that are also found in differentiated healthy tissues. The final group incorporates viral antigens and includes the protein products of human papilloma virus (ie E6 and E7 early antigens) and Epstein–Barr virus (ie EBNA). To date, the techniques we have used to identify antigens have predominantly found overexpressed antigens such as HAGE [20], PASD1 [21], SSX2IP [17, 18] and survivin [22]. Although the reason for the overexpression has not been characterised by our group, it is likely that global promoter hypomethylation, seen in many cancers, and a demonstrated cause of CTA overexpression, belies this finding.

The National Cancer Institute (NCI) has developed a well-vetted ranked prioritised list of cancer vaccine target antigens based on pre-defined and pre-weighted objective criteria, using an analytical hierarchy process. The criteria in descending order are therapeutic function, immunogenicity, role of the antigen in oncogenicity, specificity, expression level and percent of antigen-positive cells, stem cell expression, number of patients with antigen-positive cancer, number of antigenic epitopes and cellular location of antigen expression [23]. This has helped ensure funding bodies such as the National Cancer Institute can focus their resources on prioritised antigens, such as (and of particular relevance to B-ALL) survivin, Wilms’ tumour protein (WT1) and BCR–ABL, to enable the progress of immunotherapy in a directed manner. However, Cheever et al. [23] noted that no single antigen (of the 75 representative antigens that were compared and ranked) held all of the prioritised characteristics. We have noticed that there are large differences in the frequency of antigen expression between solid tumours and haematological malignancies. For example, PASD1 expression was found in acute myeloid leukaemia (AML) [21] and diffuse large B-cell lymphoma [24] but has not been found in solid tumours such as basal cell carcinoma [25] or ovarian cancer [26]. Thus, for malignancies where few antigens for treatment are known it seems appropriate to continue to look at antigen expression to identify new targets for therapy, which can also provide novel insights into the underlying disease process. We focus on presentation/diagnosis/pre-treatment samples to minimise the risk that the immune system has become compromised by treatment and have found this provides a historical view of anti-tumour immune responses developed by B [21] and T cells [27].

## Techniques for antigen identification

Cancer immunomics encompasses the determination of tumour-relevant autoantibodies and their cognate antigens [28]. We have used several techniques to identify antigens in AML, colon cancer, ovarian cancer and B-cell ALL. These techniques examine patient samples through the analysis of RNA using RT-PCR [20], qPCR [22, 29] and expression microarrays [30, 31], urine [32], or peptides presented on MHC, using mass spectrometry [33], antibodies, i.e. serological analysis of recombinant cDNA expression libraries (SEREX) [21, 24] and protein microarrays [34] and we have prioritised epitope specific T-cell responses using peptide-MHC arrays [27]. Other techniques are available, and as yet untested by our group including the next-generation sequencing (NGS) [35], RNA-sequencing (RNAseq), serological proteome analysis (SERPA), T-cell cloning and the combinatorial approach [32] and there are, of course, advantages and disadvantages to each, which primarily focus on costs, skills and equipment required.

After the identification of antigens, downstream processes are required to validate the sensitivity and specificity of the target for the treatment of diseased cells. To this end, we often use qPCR [22, 29], immunolabelling and flow cytometry [16, 22], gene expression analysis [30, 31], and peptide–MHC arrays [27] to characterise antigen expression, and epitope-specific T-cell responses.

## Identification of new targets for the immunotherapy of B-ALL

Boullosa et al. [22] examined the expression of 12 tumour antigens (BCP-20, END, G250, HAGE, NY-ESO-1, PASD1, P68 RNA helicase, SSX2, SSX2IP, survivin, tyrosinase and WT1) in 11 adult B-ALL patient samples. We found that only survivin and WT1 were expressed in B-ALL, as detected by RT-PCR (7/11 and 6/11 patient samples, respectively) but not healthy donor samples (0/8). QPCR showed survivin was the only antigen with significantly higher transcript levels in ten B-ALL patients compared to four healthy donor controls (*p* = 0.015). Immunolabelling demonstrated SSX2, SSX2IP, survivin and WT1 protein were present in all ten B-ALL samples examined but only survivin was restricted in its expression to tumour samples and was not found in healthy volunteer leukocytes.

Survivin functions as an inhibitor of apoptosis and regulator of the cell cycle. It has been found to be expressed at low levels in a number of terminally differentiated healthy tissues, with expression mostly limited to embryos and cell lines dependent on the cell cycle. In contrast, survivin expression has been reported in many tumour types, particularly lung, breast and ovary, where non-cell cycle mechanisms are driving survivin gene expression. In these cases, survivin expression is promoted by the upregulation of PI3k/AKT, JAK/Stat-3 and/or TCF–β-catenin pathways in response to growth factors or the downregulation of tumour suppressor genes such as p53, adenomatous polyposis coli (APC) and fragile histidine triad (FHIT) gene. Survivin plays a role in the inhibition of apoptosis, decreases in cell death, resistance to chemotherapy and is associated with tumour aggression. A number of clinical trials are underway that target survivin and are overviewed by Garg et al. [36], with our recent results suggesting a future for survivin as a target for the treatment of adults with B-ALL.

**Fig. 1:**
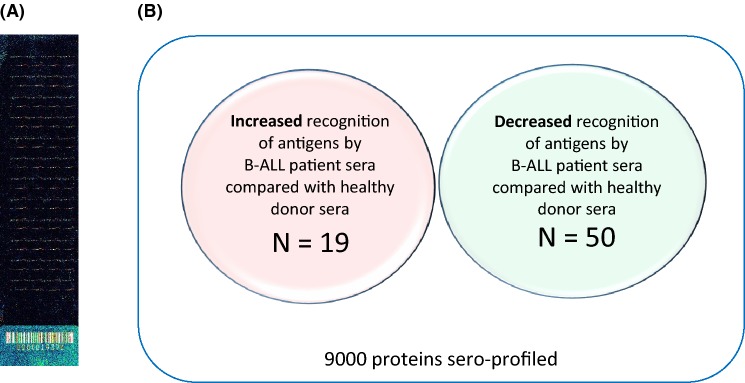
Protein microarray analysis of sera from B-ALL patients and healthy donors was used to demonstrate which antigens were more frequently recognised by healthy donor or patient sera. **a** A representative protein microarray slide after immunoscreening with 10 ul of patient sera and analysis on the ScanArray Xpress while, **b** a Venn diagram summarising the number of antigens that were significant in their recognition by patient versus healthy volunteer sera (*p* ≥ 0.02). We were particularly interested in antigens that were preferentially recognised by patient sera as these are likely to provide targets for immunotherapy, however antigens with differential recognition by patient versus healthy donor sera also provided unique insights into the biological processes that underlie adult B-ALL

**Fig. 2:**
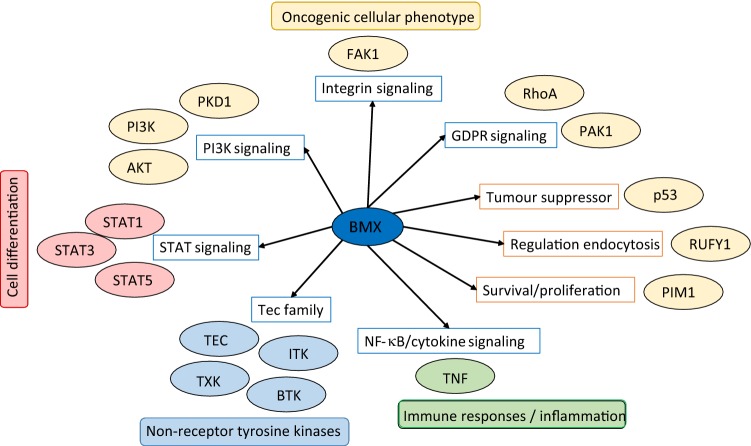
Key interactions of BMX and its pathways. The interactors by which BMX can mediate these pathways are indicated next to the pathway. The interactors and pathways that are involved in the oncogenic cellular phenotype are coloured yellow; those involved in cell differentiation are coloured red; those involved in the immune response and/or inflammation are coloured green; while those coloured in blue are the members of the Tec family of non-receptor tyrosine kinases. TEC encodes the angiopoietin-1 receptor, ITK gene encodes interleukin-2-inducible T-cell kinase; TXK encodes tyrosine-protein kinase protein and BTK encodes Bruton’s tyrosine kinase

We have also sero-profiled ten B-ALL samples [34], seven of which were pre-treatment/diagnosis, using 9000 proteins printed onto protein microarrays. We identified 19 antigens that were significant in their increased recognition by patients compared with eight age- and sex-matched healthy donor sera (*p* < 0.02) (Fig. 1). Literature searches and STRING analysis led to the prioritisation of three antigens for further investigation and bone marrow tyrosine kinase (BMX) as a target for existing therapy in clinical trials. BMX (also known as epithelial and endothelial tyrosine kinase; ETK) is located in chromosome region Xp22.2 and was first isolated by Tamagone et al. [37] in bone marrow cells. It is a non-receptor tyrosine kinase and member of the Tec kinase family. The Tec family kinases are the second largest group of non-receptor tyrosine kinases and are important in a variety of signal transduction processes (Fig. 2) [38]. BMX is expressed in hematopoietic cells of the myeloid lineage and in contrast to other members of the Tec family, which are predominantly hematopoietic cell specific; BMX is also expressed in epithelial and endothelial cells. BMX overexpression has been reported in a number of cancers, including AML, prostate cancer (reviewed in [39]), non-oncological diseases [40, 41], skin keratinocytes and upregulated during stress [42].

Considering that the B-cell receptor-activating pathway is crucial for B-cell development and maintenance, it is reasonable to expect that a TKI that targets BMX could be used to treat B-ALL. Indeed BMX is an attractive therapeutic target that has been shown to be a target for kinase inhibitors including the epidermal growth factor receptor (EGFR) inhibitor, CI-1033, that can inhibit BMX activity at a sub-micro-molar level [43]. BMX-IN-1 has been shown to inhibit BMX catalytic activity and decrease BMX protein levels in prostate cancer cells [44] while Guo et al. [45] developed the small molecule inhibitor CTN06 that can induce autophagy and apoptosis, inhibit the growth and migration of prostate cancer cells and downregulate oncogenic-related genes. Ibrutinib, is a covalent inhibitor of the Bruton’s tyrosine kinase (BTK) pathway [46] that BMX is a party to. Taken once a day as an oral dose, Ibrutinib induces apoptosis of B cells and has been shown to significantly prolong progression-free survival and OS in Phase I, II and III clinical trials (summarised in [47]). In an uncontrolled Phase I/II multicentre clinical trial, 61 patients with relapsed CLL were assessed for safety and efficacy [48]. No dose-limiting toxicity was observed and at median follow-up (14.3 months), the response rate was 95%. Similar, high response rates, especially for a single agent, were also seen in patients with R/R mantle cell lymphoma who achieved a 68% response rate in all patients, 47% of whom had a partial response and 21% had a complete response [49]. This outcome was especially notable when considering the favourable toxicity profile, durable responses and those patients who had a response included those with unfavourable risk factors.

## Microarray analysis

One of the issues when trying to analyse a meaningful number of B-ALL patient samples is its relative rarity. The way we used to circumvent this was meta-data analysis—in our case, analysing a publically available gene expression array datasets such as GSE38403 generated by Geng et al. [50]. We were interested in the expression of antigens in B-ALL patients compared with normal donors and their correlation with grade and cytogenetic abnormalities. Geng et al. [50] had performed DNA methylation and gene expression profiling on peripheral blood and bone marrow samples from a cohort of 215 adult patients with B-ALL, prior to treatment, enrolled in a Medical Research Council (MRC) UKALLXII/Eastern Cooperative Oncology Group (ECOG) (ECOG E2993) single phase III clinical trial and compared the samples to healthy donor pre-B or pro-B cells (CD19^+^ and VpreB^+^) that they had isolated by flow cytometer sorting. Of the twelve antigens examined in our Boullosa et al. [22] study, we found that only survivin was significantly overexpressed in B-ALL patient samples (*n* = 215) but not in healthy donor B-cell controls (*n* = 12) (*p* = 0.013).

**Fig. 3:**
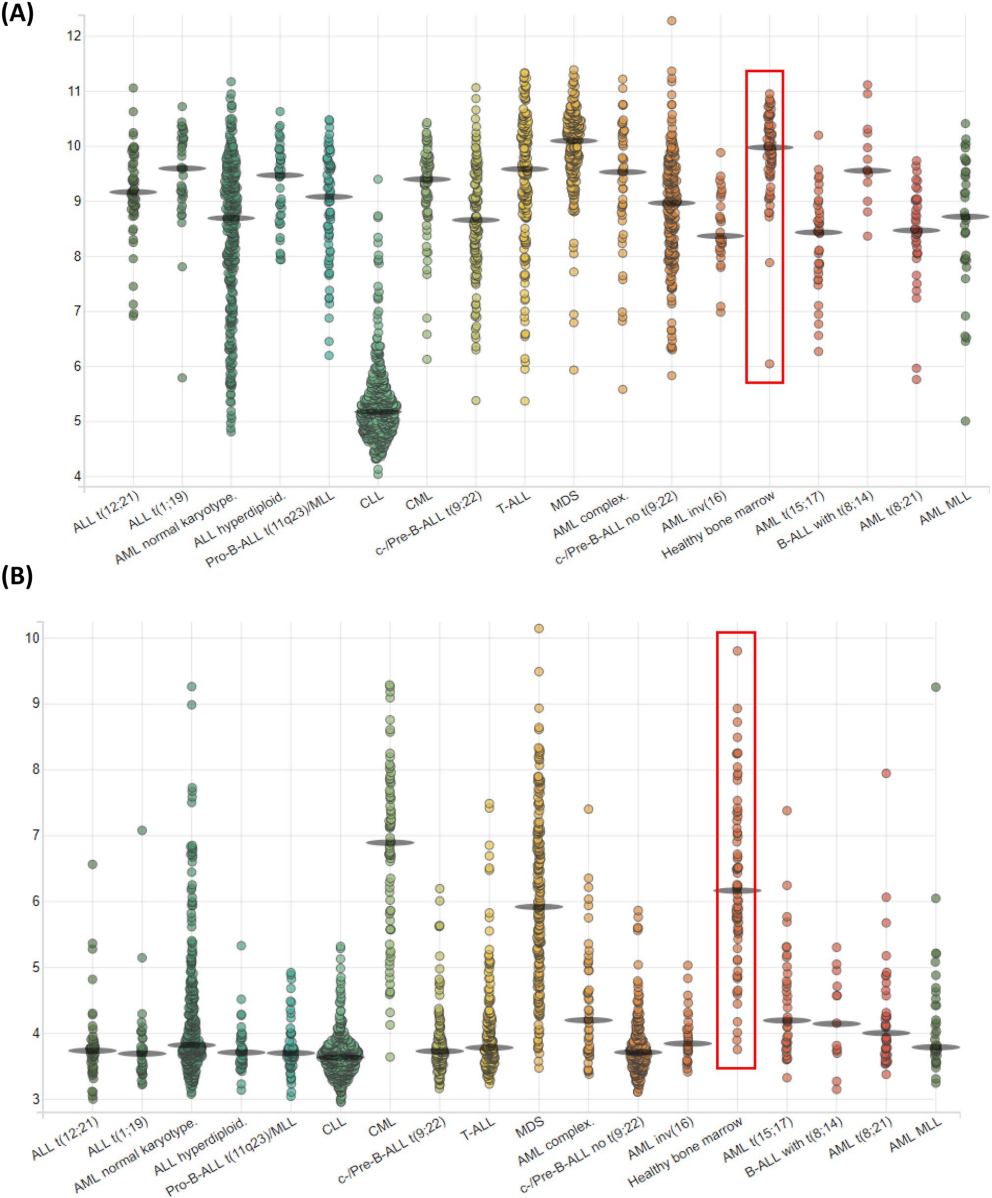
mRNA expression analysis from AML, ALL and preleukemic stages when compared to healthy bone marrow in the leukaemia MILE study. Transcripts of (**a**) survivin were decreased with high significance compared with healthy bone marrow (*p* < 0.001) in all patient groups except MDS and B-ALL with *t*(8;14) which were not significant, ALL t(1;19) which was significant (*p* < 0.05) and ALL complex and T-ALL which were significant to *p* < 0.01; **b** BMX was significantly lower with *p* values of < 0.001 for all groups compared with healthy bone marrow except c-/Pre-B-ALL *t*(9;22) (*p* < 0.05), and MDS (not significant). Cells were from GSE13159 and the data shown was generated by BloodSpot [52]. *Y*-axis indicates log_2_ expression. Each circle represents one sample analysed and the red box on each graph indicates the expression of the antigen in healthy bone marrow

Survivin has been shown to be a poor prognostic marker in a number of cancer types (recently reviewed in [51]) but we found no correlation between above or below median levels of expression and patient survival in our study [22]. Indeed, the only other notable association between survivin expression was with different 11q23/MLL abnormalities that occur in around 10% of adult ALL, and appear to have little impact on patient survival (having them versus not having them). Indeed none of the twelve antigens we initially examined [22] showed a correlation between above median expression and OS or EFS. The closest to achieving significance was SSX2IP with an association with OS at a *p* value of 0.078. This was notable as SSX2IP is also a biomarker of survival in AML [18]. Patients with above median levels of SSX2IP, at disease diagnosis, and no cytogenetic abnormalities, had improved survival *p* = 0.007 perhaps reflecting an association between elevated leukaemia-associated antigen expression at disease diagnosis and immune surveillance post-chemotherapy leading to (more) effective anti-tumour responses. However, we did find a statistically significant increase in survivin expression in normal pre-B cells compared with B-ALL patient samples at diagnosis (*p* = 0.015); in normal pre-B cells compared with B-ALL patients with cytogenetic abnormalities (*p* = 0.004); in normal donor pre-B cells compared with B-ALL patients who were BCR–ABL^+^ (*p* = 0.0031) and in normal donor pre-B samples compared with B-ALL patients with an MLL_AF9 translocation (*p* = 0.024). Decreased levels of survivin were also found in all leukaemias examined when compared to healthy bone marrow [52] (Fig. 3a) but particularly CLL (*p* < 0.001), with the exception of myelodysplastic syndrome (MDS) and B-ALL with *t*(8; 14) which showed no significant difference in survivin expression levels when compared to healthy donors. This contrasts with our expectation that survivin would have elevated expression in cancer cells and contribute to their failure to apoptose and show chemoresistance.

BMX was also found to have significantly decreased expression in all ALL groups examined in the Leukaemia Microarray Innovations In LEukemia (MILE) study and accessed through BloodSpot [52] (Fig. 3b). This decrease in mRNA transcripts was also seen in CLL patient samples and correlates with previous studies that have shown BMX expression in neutrophilic granulocytes, CD34^+^ cord blood progenitor cells, AML and chronic myeloid leukaemia patient samples but not ALL samples [53]. However, results with the TKI inhibitor, Ibrutinib, suggests that the aberration of the B-cell receptor pathway is still targetable in these patients, even if it does correlate with significantly decreased BMX transcription (Fig. 3b), and an unexpected but significant increase in BMX immunogenicity (Fig. 1b).

## Conclusions and future directions

B-ALL is a polyclonal disease with acquired specific genetic alterations that affect the occurrence of therapy resistance, treatment failure and disease relapse. Extensive attempts have been made to define the genetic basis of ALL and to identify the many genetic lesions that contribute to leukaemogenesis. In this regard, it was interesting to note that B-ALL showed decreased heterogeneity compared with healthy volunteers by principal component analysis (PCA) in our study [34].

Our identification of survivin expression in B-ALL [22] provides a new target for future therapeutic strategies that are already under investigation such as antisense inhibitors such as LY2181308, small molecule inhibitors such as YM-155, EM-1421, GDP366 and FL118, and siRNA. In addition, survivin appears to be an appropriate target for immunotherapeutic strategies such as DNA, dendritic cell and peptide vaccines (already reviewed in [36, 51]). Immunotherapeutic strategies, such as those targeting survivin, would complement the existing use of immunotherapy for patients with adult B-ALL who often receive DLIs to boost their allogeneic transplants and enhance survival.

In addition data from our proto-array studies support the use of an existing therapy (Ibrutinib) which is already in Phase III clinical trials for CLL and small lymphocytic lymphoma (SLL) [47, 48] for adults with B-ALL. Recently, Kim et al. [54] have demonstrated that pre-BCR^+^ ALL cell lines are exclusively and exquisitely sensitive to Ibrutinib treatment and that in mouse xenograft models of pre-BCR^+^ ALL, mice who received Ibrutinib had significantly enhanced survival.

In summary, we have identified and characterised the expression of a number of tumour antigens that are overexpressed in adult B-ALL at disease presentation [22, 34]. In doing so, we have found new targets for treatment, gained insights into the molecular mechanisms that underlie B-ALL at the earliest stages of disease and determined significant correlations between antigen expression and disease stage, and cytogenetic abnormalities. Future studies will continue to determine the validity of these antigens as targets for therapy, compare their sensitivity and specificity as biomarkers, and uncover their contribution to the disease state.

## Abbreviations

ADC: Antibody–drug conjugates
ALL: Acute lymphoblastic leukaemia
AML: Acute myeloid leukaemia
BiTE: Bispecific T-cell engager
BMX: Bone marrow tyrosine kinase
CAR: Chimeric antigen receptor
CLL: Chronic lymphocytic leukaemia
CRS: Cytokine release syndrome
CTA: Cancer-testis antigen
CTL: Cytotoxic T lymphocytes
DLI: Donor leukocyte infusion
EFS: Event-free survival
HSCT: Haematopoietic stem cell transplantation
mAb: Monoclonal antibodies
MDS: Myelodysplastic syndrome
MILE: Microarray Innovations In LEukemia
MLL: Mixed lineage leukaemia
OS: Overall survival
R/R: Refractory/relapsed
PD-1: Programmed cell death protein-1
TAA: Tumour-associated antigen
TKI: Tyrosine kinase inhibitor
WT1: Wilms’ tumour protein
